# Self-Guided Web-Based Interventions: Scoping Review on User Needs and the Potential of Embodied Conversational Agents to Address Them

**DOI:** 10.2196/jmir.7351

**Published:** 2017-11-16

**Authors:** Mark R Scholten, Saskia M Kelders, Julia EWC Van Gemert-Pijnen

**Affiliations:** ^1^ Centre for eHealth & Wellbeing Research Department of Psychology, Health and Technology University of Twente Enschede Netherlands; ^2^ Optentia Research Focus Area North-West University Vanderbijlpark South Africa

**Keywords:** eHealth, review, embodied conversational agent, human computer interaction, clinical psychology, health behavior, Web-based intervention, adherence, intelligent tutoring system, ITS

## Abstract

**Background:**

Web-based mental health interventions have evolved from innovative prototypes to evidence-based and clinically applied solutions for mental diseases such as depression and anxiety. Open-access, self-guided types of these solutions hold the promise of reaching and treating a large population at a reasonable cost. However, a considerable factor that currently hinders the effectiveness of these self-guided Web-based interventions is the high level of nonadherence. The absence of a human caregiver apparently has a negative effect on user adherence. It is unknown to what extent this human support can be handed over to the technology of the intervention to mitigate this negative effect.

**Objective:**

The first objective of this paper was to explore what is known in literature about what support a user needs to stay motivated and engaged in an electronic health (eHealth) intervention that requires repeated use. The second objective was to explore the current potential of embodied conversational agents (ECAs) to provide this support.

**Methods:**

This study reviews and interprets the available literature on (1) support within eHealth interventions that require repeated use and (2) the potential of ECAs by means of a scoping review. The rationale for choosing a scoping review is that the subject is broad, diverse, and largely unexplored. Themes for (1) and (2) were proposed based on grounded theory and mapped on each other to find relationships.

**Results:**

The results of the first part of this study suggest the presence of user needs that largely remain implicit and unaddressed. These support needs can be categorized as task-related support and emotion-related support. The results of the second part of this study suggest that ECAs are capable of engaging and motivating users of information technology applications in the domains of learning and behavioral change. Longitudinal studies must be conducted to determine under what circumstances ECAs can create and maintain a productive user relationship. Mapping the user needs on the ECAs’ capabilities suggests that different kinds of ECAs may provide different solutions for improving the adherence levels.

**Conclusions:**

Autonomous ECAs that do not respond to a user’s expressed emotion in real time but take on empathic roles may be sufficient to motivate users to some extent. It is unclear whether those types of ECAs are competent enough and create sufficient believability among users to address the user’s deeper needs for support and empathy. Responsive ECAs may offer a better solution. However, at present, most of these ECAs have difficulties to assess a user’s emotional state in real time during an open dialogue. By conducting future research with relationship theory–based ECAs, the added value of ECAs toward user needs can be better understood.

## Introduction

Meta-analyses have demonstrated that Web-based interventions for mental health have become reasonably successful treatments against common mental health problems such as depression and anxiety [1-3]. However, it is a consistent finding that human-supported Web-based therapeutic interventions outperform self-guided interventions [4] (in which there is no support from a human). The mere remote presence of a human being delivering informational support, emotional support, or a therapeutic service results in significantly higher effect sizes [5]. In addition, human-supported interventions achieve higher rates of adherence; more participants use the intervention as intended, for example, by completing all the lessons of an intervention [1,3,6]. Nonadherence is an important issue in Web-based interventions for mental health [7] and becomes an even bigger problem when evidence-based therapies are deployed as free-to-access self-guided Web-based therapeutic interventions [8]. In these interventions, adherence, defined as the percentage of users who complete all lessons, falls to a level as low as 1.0% [7] or even 0.5% [8].

The higher rates of adherence in human-supported interventions can be explained in favor of therapists who do an effective job in motivating clients during their change process [5]. However, positive effects of electronic interventions have also been found by using features such as reminders and tailored advice [9]. Interestingly enough, Talbot [10] concludes in her meta-study that the involvement of a professional support provider, a therapist, is not key. Instead, a minimal level of nonguiding human contact is key. Irrespective of whether this type of contact is provided by a layperson or a professional, it has equally large positive effects on intervention adherence. Moreover, scheduling support on one’s own can already have an effect on treatment effectiveness. A telephone contact, scheduled at the start of reading a self-help book, yields surprisingly large completion rates and treatment outcomes [11]. This poses the question: what support is needed to achieve higher rates of adherence and effectiveness? A study of Cavanagh and Millings [12] provides evidence of built-in “common factors,” such as generating hope, empathy, warmth, collaboration, and feedback, that increase the effectiveness of interventions. However, there is no generally accepted definition of these “common factors.” The urgency of support is expressed by the statement of Kreijns et al [13] who declare that the reason that digital learning environments fail is because of socioemotional processes being “ignored, neglected, or forgotten.” As Web-based health interventions share many characteristics with digital learning environments, it is a fair assumption that the same socioemotional processes play a role and should be subject to study in relation to adherence.

The following challenge is how these socioemotional processes could be handled within Web-based health interventions. As suggested by Bickmore [14], putting an embodied conversational agent (ECA; also called relational agent) as an adjunct to a self-care application can be a means to support users. An ECA, according to Bickmore, is *a computational artifact designed to build long-term socioemotional relationships with users*. Within the context of health care, Bickmore [14] suggests that the ECA-user relationship can contribute to trust and therapeutic alliance for the purpose of *enhancing adherence to self-care treatment regimens*.

Altogether, ECAs hold the promise that they can bring in social, emotional, and relational elements to the user interface. It is, however, less clear to what extent ECAs can (1) truly handle *expressed needs* of users of electronic health (eHealth) interventions and (2) provide user stimulation that will truly pay out in terms of *user adherence to eHealth interventions*. This was the rationale for us to conduct the research as described within this paper. Therefore, the aim of this study was to structurally analyze existing literature to extract (1) user needs pertaining to eHealth interventions and (2) the capabilities of ECAs to fulfill these needs within the larger objective of enhancing user adherence.

## Methods

### Study Design

This study was performed by means of structured data collection within the Web of Science and Scopus databases. The scoping review was chosen as research method. A scoping review aims to map the existing literature in a field of interest in terms of the volume, nature, and characteristics of the primary research [15]. The rationale for choosing a scoping review for the subject of this paper is that research on Web-based interventions forms a large and diverse body of literature. Within this literature, the role of support and its relationship to user motivation are barely explored and poorly understood. This is equally the case for system support provided by ECAs within, for example, social learning contexts [16]. To the best of our knowledge, no studies were conducted that systematically aimed to match user needs for Web-based interventions to ECA capabilities to find potential solutions for low adherence to the interventions. Having said that, seminal studies (eg, [17]), have suggested and partly demonstrated that ECAs have the potential to stimulate and motivate users, which ultimately may have a positive effect on intervention adherence—which underscores the importance of this study.

This study is divided into two parts:

Part 1: a scoping review of meta-studies on user support in Web-based interventions. The focus of this review was on generic user support needs, irrespective of the intervention type and type of disorder.

Part 2: a scoping review of the opportunities of embodied conversational agents to deliver support within Web-based interventions for health or learning.

#### Research Question Part 1

Is there a set of generic user support needs that are currently not sufficiently addressed within eHealth interventions requiring repeated use that may result in a lower user experience and therefore lower user adherence?

#### Search Strategy Part 1: Meta-Studies on Support in Web-Based Interventions

The Scopus database was searched with a combination of the concepts “support,” “Web-based intervention,” and “review.” For each of the concepts, multiple keywords were used (see Multimedia Appendix 1). Textbox 1 provides the inclusion and exclusion criteria.

**Figure 1:**
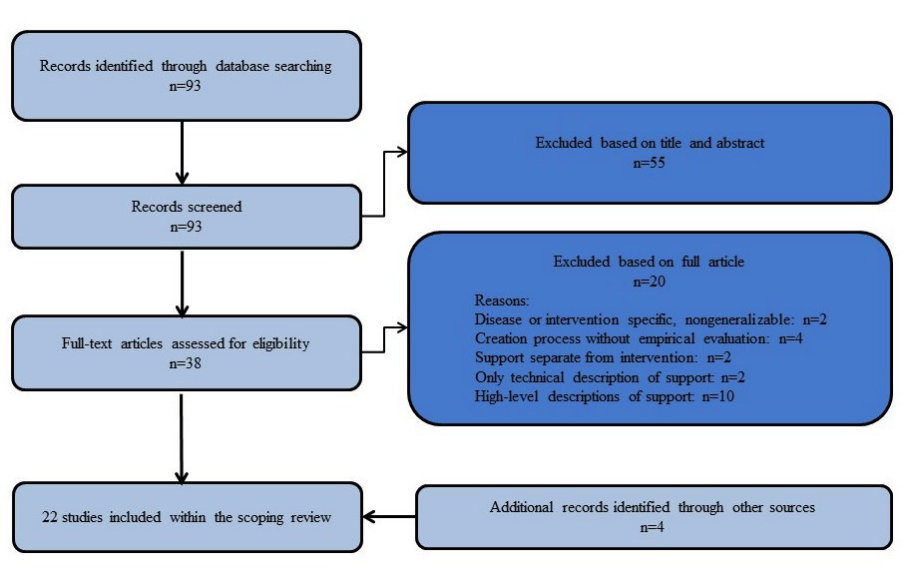
Flow diagram of the study selection of part 1 of the scoping review.

The search resulted in 93 studies. On the basis of our inclusion and exclusion criteria, we selected 18 studies. By checking the references of these selected studies, we found another 4 relevant papers. Finally, 22 papers were included. See Figure 1 for the selection process.

**Inclusion and exclusion criteria.**

Inclusion criteria were as follows:

- Papers had to address a Web-based intervention for a mental or physical disorder in which support was the subject of the study
- Papers had to review multiple interventions/studies or present ideas based on literature or an earlier study

Exclusion criteria were as follows:

- Papers that restricted themselves to a specific disease and/or intervention and did not generalize to eHealth within a broader context
- Papers that described the creation of a Web-based intervention and did not take the empirical evaluation in scope
- Papers on social media and support solutions that were studied separate from the Web-based intervention events
- Papers that did not describe support in functional terms (eg, praise, reassurance) but only in technical delivery terms (eg, short message service [SMS], email)
- Papers that analyzed Web-based interventions using high-level descriptive factors (eg, “interactive component,” “supervision,” “tailored”) without going into more detail

#### Data Extraction Part 1

The entire content, including the introduction, discussion, and references, of the 22 studies was checked from the users’ perspectives regarding usability and the needs they expressed. We applied grounded theory, applying the following phases:

Phase 1: We labeled the descriptions of user support needs in the way they were defined within each study.

Phase 2: We analyzed the various labels and categorized them.

Phase 3: We distilled main themes out of these categories with the aim of representing the user support content within the selected studies.

#### Research Question Part 2

What are main supportive features of ECAs that could potentially address user support needs?

#### Search Strategy Part 2: Opportunities of Virtual Coaches to Deliver Support Within Web-Based Interventions for Health or Learning

The search aimed to create a generic idea of the capabilities of ECAs for supportive purposes. The Scopus and Web of Science databases were searched with a combination of the concepts “embodied conversational agents,” “Web-based intervention,” and “support.” For each of the concepts, multiple keywords were used (see Multimedia Appendix 1). As ECAs are often used within an e-learning context, it was decided to include studies on intelligent tutoring systems (ITS) as well. ITS was included as a keyword within the concept of “Web-based intervention.” See Textbox 2 for inclusion and exclusion criteria.

The systematic search resulted in a limited number (8) of studies. Moreover, these studies addressed a wide range of topics, from physical attributes [18], architecture [19], route planning [20], nonverbal behavior [21], virtual museum guide [22], and empathy [23], to theoretical models [24] and articulation rates [25]. None of the systematically selected studies provided a high-level picture of the capabilities of ECAs with regard to support delivery. Therefore, it was decided to expand the number of studies by means of hand search. We started the hand search in Google Scholar by both (1) checking references and (2) further searching on terms found within the 8 selected studies.

We initiated the hand search on the following basis:

1. Finding synthesizing information on ECAs within a health or pedagogical (ie, e-learning) context with a focus on the delivery of support and motivating users. We started with the information found in [23] and additionally searched for meta-studies on ECAs.
2. Finding additional (founding) studies on the computers as social actors (CASA) effect as mentioned within [18] and [23].
3. Finding additional information on relationship building [25] and measures of relationship building as briefly described in [21,25].
4. Finding additional information on theoretical models related to ECAs as touched upon in [24].

**Figure 2:**
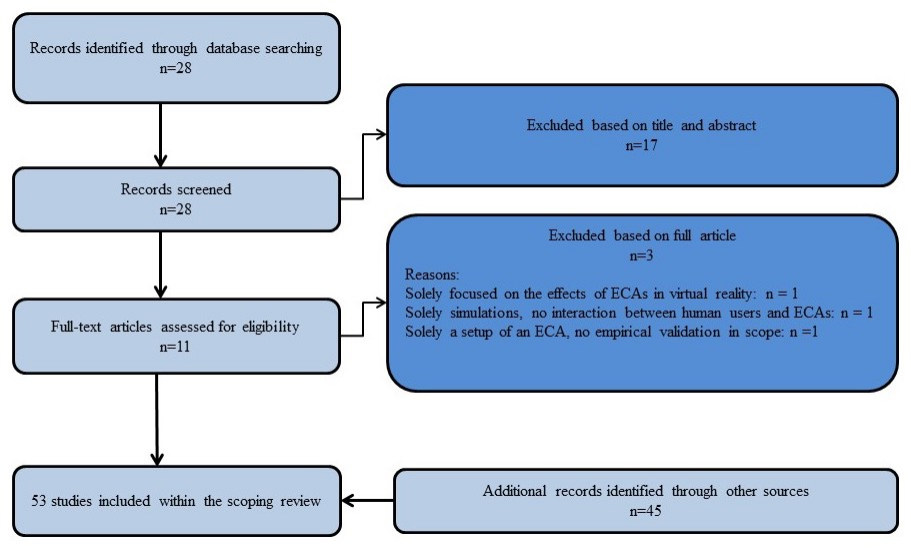
Flow diagram of the study selection of part 2 of the scoping review.

The entire search procedure resulted in including 53 studies (Figure 2).

#### Data Extraction Part 2

Using grounded theory, the entire content, including the introduction, discussion, and references, of the selected studies was analyzed with the aim of finding specific information on user support as carried out by ECAs. As this information was scarce, we decided to formulate three concepts that we thought were most relevant for eHealth and covered substantial information of the ECA literature that was semantically related to the notion of user support.

We formulated the following three concepts:

1. Which *multimedia* aspects of ECAs are relevant for eHealth environments?
2. What kind of *relationship* is applicable between ECAs and users?
3. How *useful* are ECAs for user adherence in eHealth?

Out of the three concepts we formulated, 8 themes coherently described a specific ECA topic.

**Inclusion and exclusion criteria.**

Inclusion criterion was as follows:

- Papers had to address embodied conversational agents (ECAs) interacting with users or studies on ECAs interacting with users

Exclusion criteria were as follows:

- Papers that solely focused on virtual reality
- Papers in which interaction between human users and ECAs was absent
- Papers that described the design of an ECA but did not take the empirical validation in scope

## Results

### Part 1: Results and Themes Found Within the Studies on the Need for Support in Web-Based Interventions

The 22 analyzed papers suggest that a myriad of subtle interactions between users and computers play an important role in keeping a user motivated in continuing the Web-based intervention.

We formulated 8 themes according to our data extraction procedure. We further condensed these 8 themes into 2 main need-and-support concepts that in our view summarized the subject and that would help us during further analysis.

Users expressed the need for concrete feedback on their performance. Within the literature, this need is described as the principle of closure [26]: the confirmation that an action has been successfully performed. This indicates that users of Web-based interventions could benefit from task-related interaction and support (eg, “Thank you for submitting your homework for this week. You sent it well on time.”). We call this *task-related support*.

Users expressed the need for interest and support for the issues they are dealing with. This suggests that users of Web-based interventions could benefit from emotional support that acknowledges both the user’s endeavors during the change program and the originating issue the user is dealing with. This concept was based on the literature we found earlier [12,13]. We call this *emotion-related support*.

Table 1 shows the user needs that became apparent in the included papers and that we related to the two common needs-for-support elements mentioned above.

The user needs and issues, mentioned in Table 1, are discussed in greater detail below:

#### Need 1: Overcome User Feelings of Isolation

The anonymity of Web-based interventions seems to play out both as a strength and weakness. Users feel encouraged to speak out but sometimes also feel isolated because of its anonymous nature. As formulated by McClay [31], “The findings showed that the desire for privacy and secrecy had a detrimental impact on participants’ help-seeking, their use of the intervention, and the support they could seek from family and friends” and, as expressed by Foster [41], “the difficulties of talking to others.”

Both task-related and emotion-related system support could potentially counteract feelings of isolation.

#### Need 2: Deeper Interest in the User’s Situation

Users seem to expect (and probably need) a deeper interest in their situation. Knowles et al [28] concluded the following as a shortcoming found in 7 out of 8 studies: “sensitivity to ‘Who I am’ as a patient.” Todd [32] described this as “that would deal with how to live your life as somebody who suffers from Bipolar.”

This is a case for emotion-related system support.

**Table 1:** User needs and issues and common user support mechanisms that can potentially fulfill these needs.

User need or issue	Support mechanism to fulfill the need	Source that describes the support mechanism
1. Overcome users’ feelings of isolation	*Task-related support*^a^ can conform to this need by setting and reviewing log-in goals, positively reinforcing log-in and site use and answering questions regarding the functionality of the site.	[6,27-30]
	*Emotion-related support*^b^ can satisfy this need by establishing a supportive relationship. In case log-in goals are not met or any other sign of diminished use of the intervention appears, the system can intercede and encourage the use of the Web-based intervention.	[6,27,29,30]
2. Deeper interest in the user’s situation	*Emotion-related support*^b^ can fulfill this need by providing the users with the opportunity to talk about the impact of the disease on their life.	[28,31,32]
3. Interest in fundamental daily issues the user is struggling with	*Emotion-related support*^b^ can meet this need by asking the users about their daily experiences and issues and by subsequently providing acknowledgment. If the user expresses a need for practical advice, the system could provide it accordingly or refer to a nurse or doctor connected to the system. This can be considered as *task-related support in a broader context*.	[28,33]
4. The ability for the user to refine the communication process	*Emotion-related support*^b^ provided alongside a more open interaction between user and system (eg, by means of bidirectional free text or free speech) could potentially (and so far theoretically) increase the user’s feeling of contributing to his/her own change process.	[34,35]
5. The user’s need for encouragement	*Emotion-related support*^b^ could be delivered in terms of praising the user or delivering rewards or by other types of encouraging behavior.	[6,27,32-34,36-39]
6. Performance feedback mechanism for user responses	*Task-related support*^a^ can render this need by providing corrections in case the user made factual errors (eg, homework with factual information about an illness). In addition, preferably if opted so by the user, the user’s achievements can be plotted against the achievements of the user’s peer group. This scenario is applicable especially for user performances that can be measured in physical terms such as step counts.	[6,36,40-42]
7. Users coping with experiences of negative affect during their change process	*Emotion-related support*^b^ can provide a dose of positive affect in case a phase of negative user affect that merits such a dose could be reliably distinguished.	[43]
8. Creating a setting of accountability toward the user	*Task-related support*^a^ can play a positive role by objective goal setting, measuring the goals set, reminding the user of their goal set, and indicating which of these goals have (not yet) been met.	[29,30,33,38,39,44-47]

^a^Task-related support: the confirmation that a user action has been successfully performed.

^b^Emotion-related support: acknowledgement of both the user’s endeavors during the change program and the originating issue the user is dealing with.

#### Need 3: Interest in Practical Daily Issues the User Is Struggling With

Users seem to wish for a form of deeper interest in their practical daily issues. This need is described by Knowles et al [28] as “sensitivity to ‘How I Feel’, recognizing the demands of depression on the user (such as emotional and motivational difficulties, and problems with concentration),” and reported on by Todd [32], “In terms of practical issues participants described wanting support understanding their legal rights, managing debt, managing pregnancy and coping with seasons and time zones.”

This is a case for emotion-related system support. In case the user requests practical advice for daily issues, task-related support on these issues can also contribute.

#### Need 4: The Ability for the User to Refine the Communication Process

As mentioned by Donkin [34], users filling in questionnaires about how they felt said that the questionnaire did not cover their feelings. Subsequently, these users had a strong wish to contextualize their answers. McClay [31] described this as “the thought of having to fill more books in and logs.”

A noninteractive tool as a questionnaire is perfectly fit for structurally gathering experimental user data. However, it may be less appreciated as it “forces” the users to answer according to its rigid structure. Emotion-related system support provided alongside a more open interaction between user and system (ie, by means of bidirectional free text or free speech) could potentially (and so far theoretically) increase the user’s feeling of contributing to his or her own change process.

#### Need 5: The User’s Need for Encouragement

As noted by Donkin et al [34] and as quoted by Mohr [30], “patients want feedback on whether they are on the ‘right track’ in their web-based intervention.” Foster [41] described this as “Users are encouraged to set weekly SMART goals on the basis of the content of these sessions.”

Encouraging users during the intervention can likely be achieved by emotion-related system support.

#### Need 6: Performance Feedback Mechanism for User Responses

Somewhat comparable with the statement of Donkin et al [34], Helgadóttir [42] describes that many CCBT (computerized cognitive behavioral therapy) programs would benefit from a performance feedback mechanism for user responses. This would expand the system’s ability to direct the users during their change program. Gorlick [44] reported on this as “participants reported that if they were to spend time answering the questionnaires, they would prefer feedback about their responses.”

Providing a direct task-related response such as “I have received your answers, thank you for your time and effort. Please allow me to comment on your answers” would immediately acknowledge the user’s effort invested. By later analyzing the user responses and by providing feedback via email, a second, more profound task-related support mechanism could be implemented.

#### Need 7: Users Coping With Experiences of Negative Affect During Their Change Process

As formulated by Todd [32], “Participants struggled to understand why they feel this way and desired to know why lack of motivation happens and how to overcome it.” Kraft et al [43] suggest that individuals should be assisted in coping with experiences of negative affect during their change process. They make a claim that many change program users struggle with the tension between their aspirations and their actual status and behavior. During this struggle, the client’s internal process of self-regulation is activated to alleviate the tension. Too much burden on the self-regulation process leads to ego depletion [43], a status of a low level of mental energy. This status often results in increased relapse vulnerability and potentially therapy nonadherence. As a way to reverse this ego-depletion process, Kraft et al [43] recommend a dose of positive affect, next to a period of rest for recovery. Emotion-related system support could provide such a dose of positive affect. The challenge would be to determine the moment that ego depletion is close.

#### Need 8: Creating a Setting of Accountability Toward the User

As described by Bradbury [33], “Some participants’ accounts of coaching suggested that they experienced accountability to the coach, which made them more committed and motivated.” Mohr et al [39] also stress the importance of creating a setting of accountability toward the user, “the implicit or explicit expectation that an individual may be called upon to justify his or her actions or inactions.”

For such a setting, certain preconditions are necessary, such as participants who understand and agree with the benefits of their expected future behavior. Other preconditions are concrete goal setting and performance monitoring. Task-related machine support can play a positive role by reminding the users of their goal set and by indicating which of these goals have (not yet) been met. Note that accountability might be harder to trigger among users who have been assigned to health interventions by their doctors and who did not primarily opt to participate by themselves.

### Part 2: Results and Themes Found Within the Studies on ECAs With Motivational Capabilities

Table 2 shows the results and the themes that were found in the selected studies. Out of 8 themes found, 7 themes originate from the literature found during the systematic search and were bolstered during the hand search. Theme 8, methodological issues, was uniquely based on hand search information.

#### Theme 1: Computers as Social Actors

A large body of studies on ECAs refer to the CASA effect [49,50,51] as a cornerstone for studying human-computer interactions and especially human-ECA interactions. The CASA effect demonstrates that humans treat media—in some respect—in the same way as they treat fellow humans. Various manifestations of this effect have been described as follows:

- Computers that display flattery texts toward their users are preferred by their users compared with computers that do not display such texts.
- Computers that textually praise other computers are better liked than computers that praise themselves, and computers that *criticize* other computers are disliked compared with computers that criticize themselves.
- Users who are partnered with a computer on basis of a color (eg, the blue team) will have a more positive opinion about the computer and cooperate more with it than users who have to partner with a computer of the opposite, differently colored team.

As an explanation of the CASA effect, it has been proposed that humans have a strong innate tendency to make social connections with other humans and other living creatures such as pets. This human tendency becomes real when objects such as personal computers (PCs) demonstrate activities that could be socially interpreted by their users [51]. Although PCs can act socially, human users are logically aware of their nonsocial and nonliving status. This seems a paradox: why would a human user socially respond to a PC while at the same time realizing that a PC does not warrant it? Nass and Moon [49] refer to “mindless” (automatic, largely unaware) human behavior that the machine can trigger. This mindless behavior will be displayed as long as it remains socially acceptable. This phenomenon is also associated with the notion of “suspension of disbelief,” meaning that up to a certain point humans are willing to apply social rules to nonhuman yet communicative objects, irrespective of their nonliving status.

**Table 2:** Themes on supportive embodied conversational agents.

Theme	Explanation	Sources
1. Computers as social actors	Humans treat media in the same way as they treat other humans.	Systematic search: [23] Hand search: [48-51] (concept=relationship)
2. Open dialogue between user and computer	Embodied conversational agents (ECAs) have the ability to have an open verbal dialogue with users.	Systematic search: [22] Hand search: [52-54] (concept=multimedia)
3. Visible conversational partner	Interaction with a “talking face” leads to more trust and believability.	Systematic search: [18,20,23,24] Hand search: [55-65] (concept=multimedia)
4. Human-ECA relationship	Interactions with an agent can lead to a relationship, which is important to keep users engaged over time.	Systematic search: [25] Hand search: [17,66-72] (concept=relationship)
5. Measures of the human-ECA relationship	Human-ECA relationship quality can be measured.	Systematic search: [17,21,68,73] (concept=relationship)
6. Responsive verbal and nonverbal communication	Computers should have the ability to notice and respond to verbally and nonverbally expressed emotions from their user to create a more natural interaction.	Systematic search: [23] Hand search: [63,74-81] (concept=relationship)
7. Impact of ECAs on user motivation	There is evidence that ECAs can motivate users, which is highly dependent on ECA implementation, context, task, etc.	Systematic search: [19] Hand search: [57,74,82-84] (concept=useful for eHealth^a^)
8. Methodological issues within ECA research	Most experiments into ECAs face similar methodological issues, which have to be taken into account when interpreting the research.	Hand search: [85-89] (concept=useful for eHealth^a^)

^a^eHealth: electronic health.

#### Theme 2: Open Dialogue Between User and Computer

The theme that follows is the ability of computers and ECAs to have an open verbal (textual or speech) dialogue with users. Within regular, day-to-day human-computer interaction events, a user who interacts with his or her information technology system will typically activate predefined menu options such as the “save as” option. Subsequently, the computer will respond to the request by presenting a pop-up window, which will enable the user to type in the file name of the document. In such a closed dialogue scenario, the interactions between the user and the software traditionally have a task-specific character (ie, serve to reach the specific goal of saving a document), have a short duration, and are typically initiated by the user (and not by the computer). In contrast, ECAs enable more open-ended and more relationship-oriented interactions. Interactions between ECAs and users can span multiple question-and-answer pairs and can therefore be interpreted as a dialogue.

The ELIZA (software created by Joseph Weizenbaum at the MIT Computer Science and Artificial Intelligence Laboratory Cambridge, MA, USA) study [54] described an early version of a textual psychotherapist that gave “canned” responses to user questions as a result of quickly processing the input text provided and create a response out of it without realizing what the user had said (eg, a question such as “Eliza, I feel miserable today” and an answer “How often do you experience feelings of being miserable?”).

Later studies create richer dialogue contexts to explore the capabilities of computers interacting with humans. One of the examples is a study that has shown that a robot taking the role of a museum guide who uses, for example, empathy and humor in his conversation style led to a more positive attitude toward the robot than the same robot without this enhanced conversation style [22]. A second study showed that an ECA with high-dialogue capabilities reached more accurate answers when interviewing a subject than an agent with less dialogue capabilities [52]. A third study [53] aimed to explore where open-dialogue options between users and ECAs would lead to. The authors report that when learners are given opportunities to guide an open conversation, they especially ask off-topic questions. For example, learners often want to know about the agents’ operating systems, design, purpose, and capabilities. Such conversations seem to serve the “testing” of agents’ abilities during which learners are attempting to discover the boundaries, limits, and capabilities of agents through “game-like” inquiry.

**Table 3:** Main theories and effects of visible embodied conversational agents.

Embodied conversational agent (ECA) theory	Explanation	Source
Theory of social inhibition/facilitation	When in the presence of others, people perform learned tasks better and novel tasks worse. Empirical results have demonstrated that this principle also applies for the presence of ECAs.	[65]
Social agency theory	By adding a visible ECA as a screen tutor, the social interaction schema is primed, which will cause the learner to try to understand and deeply process the computer-delivered instructions.	[61]
Social modeling/social learning theory	Humans derive their knowledge, attitudes, behavior, and goals by observing and imitating the surrounding social agents.	[18,24]
Situational dependency	Pedagogical agents are helpful when there is a need to increase companionship and decrease complexity.	[57]
Social exchange theory	People prefer equitable relationships in which the contribution of rewards and costs are roughly equal. This equity principle also applies to human-computer relationships.	[58]
Persona effect	The presence of a lifelike character in an interactive learning environment—even one that is not expressive—can have a strong positive effect on a student’s perception of his or her learning experience.	[59]
Image principle	The image of an ECA is not a key factor for learning; instead, the level of animation of the ECA is the key factor for learning.	[61]

#### Theme 3: Visible Conversational Partner

The next theme is the visibility of the conversational computer depicted as a (either static or animated) human face. According to Lisetti [60], the human face has a special status in human-to-human communication as it has often been identified as the most important channel for conducting trust and believability. As Lisetti states, the face as a communication channel has a higher status than bodily regions such as posture and gesture [56]. Multiple studies have supported this notion by demonstrating that users preferred to interact with a “talking face” instead of a text-only interface [64], an anthropomorphic agent together with a human voice has led to greater agent credibility [55], and visible agents have led to greater positive motivational outcomes [63] and task performance [65].

Besides empirical research, there are multiple theories that support this notion. The theories that were mentioned in the included sources are listed and explained in Table 3.

Despite these positive experimental results and theoretical support for a visible, human-like PC, the visibility subject is somewhat controversial. Strong claims against the human face are provided by Norman [26] by his statement that a human face triggers false mental models and thus creates wrong user expectations. Other critique is provided by Rajan et al [62] who demonstrated that it is first and foremost the voice (and not the visibility of the ECA) that is responsible for positive learning effects.

#### Theme 4: Human-ECA Relationship

The fourth theme is the concept that regular human-computer interaction events result in a relationship. Routine interactions between a user and his or her computer should be regarded as contributions to this human-computer relationship, as is argued by Bickmore et al [17]. Although this relationship may be implicit, it has an impact on the user. The relationship plays a role even in case no relationship skills (eg, empathy, humor) have been designed and built into the machine.

The question arises whether an ECA with a relationship-focused design could behave and be perceived as a competent social actor. This quality of the ECA as a conversational partner is impacted by the following:

- *Interaction duration*. As described by Krämer et al [71], getting people engaged with ECAs is easy, but keeping them engaged over time is much more challenging. Bickmore et al [17] (on physical activity) and Creed et al [67] (on fruit consumption) conducted emotional virtual coach studies that spanned more than 28 days. They both found that deploying the emotional ECA did not result in user behavior changes but that users in general preferred to interact with the emotional virtual coaches.
- *Natural versus forced interaction*. Gulz [69] suggests that most ECA studies force the human-computer relationship too much. Users have no other option than to interact with the ECAs they are confronted with.
- *User personality*. Von der Pütten et al [72] make clear that it depends on the personality of the user how the human-computer relationship will develop. They demonstrated that five user personality factors were better predictors for the evaluation outcome of ECAs than the actual behavior of the ECA.

#### Theme 5: Measures of the Human-ECA Relationship

The literature found mentions two regular measures with regard to the human-ECA relationship.

##### Measure 1: Working Alliance

Working alliance is a construct that originates from the psychotherapy literature and has been described as “the trust and belief that the helper and patient have in each other as team-member in achieving a desired outcome” [73]. Bickmore et al [66] applied the working alliance inventory in their 30-day longitudinal study with an ECA acting as an exercise coach. Participants who interacted with an ECA that was relational behavior–enabled (empathy, social chat, form of address, etc) scored the ECA significantly higher on the working alliance inventory compared with participants who interacted with the same ECA with the relational behaviors disabled.

##### Measure 2: Rapport

A second important human-computer relationship measure is rapport. Rapport has been described as “the establishment of a positive relationship among interaction partners by rapidly detecting and responding to each other’s nonverbal behavior” [68]. Measurement of rapport has been conducted by Gratch et al [68] in their evaluative ECA study. Their results showed that the experience of rapport was of a comparable level compared with a face-to-face (ie, human interlocutor) condition.

#### Theme 6: Responsive Verbal and Nonverbal Communication

Within human-to-human communication, the exchange of nonverbal information plays a key role. Social psychologists assert that more than 65% of the information exchanged during a person-to-person conversation is conveyed through the nonverbal band [75,81]. The nonverbal channel is said to be especially important to communicate socioemotional information. Socioemotional content [76] is vital for building trust and productive human relationships that go beyond the purely factual and task-oriented communication. D’Mello et al [76] describe the mutual impact of user and (synthetic) computer emotions as an affective loop, which is pictured as follows:

- The user first expresses his or her need and accompanying emotion through verbal and physical interaction with the machine, for example, through detectable gestures, usage of the keyboard, or spoken language.
- Then, the system generates an affective reply, through words, speech, and animation with the intention to respond to the user’s need.
- This response affects users in such a way that they become more involved in their further interaction with the computer.

Others, such as Doirado [78], use the term “belief, desire, and interest” (BDI) in relation to a system that is (to some extent) capable of assessing the user’s needs.

Concerning the importance of the affective loop and BDI, there are 2 stances:

- *Stance 1: Responsiveness of ECAs (affective loop; user BDI capable system) is a critical condition for prolonged user interaction.* Doirado et al [78] confirm the importance of the affective loop mechanism and state that an ECA that lacks the capacity to assess the user’s BDI and to conform to the user’s needs by adapting its behavior (a nonresponsive ECA) will break the user’s suspension of disbelief.
- *Stance 2: Autonomy of ECAs (no affective loop; system is unaware of the user’s BDI) is a sufficient condition for prolonged user interaction.* Rosenberg-Kima et al [63] deployed an autonomous (ie, nonresponsive) ECA that introduced itself and provided a 20-min narrative about 4 female engineers, followed by five benefits of engineering careers. The ECA was animated and its voice and lip movements were synchronized. The ECA acted autonomously; interaction between participants and ECA was purely restricted to the user clicking on the button for text topic. The results showed that the self-efficacy of the users and of their interest in the subject presented was significantly higher within the ECA + voice condition compared with the voice-only condition. In support of these results, Baylor et al [55] state that people are willing to interact with anthropomorphic agents even when their functionality is limited. As she indicates, the mere visual presence and appearance will in some contexts be the determining factor and not so much its supportive, conversational, or animation capabilities.

#### Theme 7: Impact of ECAs on User Motivation

Meta-studies and reviews [69,81,85,86,89] have reported on claims and evidence for positive ECAs’ effects on learning, engagement, and motivation.

Schroeder et al reviewed 43 studies and concluded that pedagogical agents have a small but significant effect on learning as ultimate outcome. Within their study, Schroeder et al [81] did not make a distinction between responsive and nonresponsive ECAs. Specific research with regard to motivating users has also been conducted by deploying responsive ECAs with the task to notice user frustration and to empathically respond to it. Autonomous delivery of warmth and empathy by ECAs toward users has shown positive effects, and studies show that this effect may be larger at the time the user experiences frustration [74,86,88].

Altogether, the evidence for ECAs capable of motivating users is inconclusive. ECAs, whether they are nonresponsive or responsive, provide a positive user experience as a result of their entertainment capabilities. Responsive ECAs when specifically designed to detect user frustration and to empathically respond to it have also empirically demonstrated positive effects on user attitudes. However, these positive effects have not yet been found in ecologically valid contexts. Instead they were found within constrained contexts such as games with clear win-and-lose rules and as a result of deliberately induced user frustration.

#### Theme 8: Methodological Issues Within ECA Research

The inconclusiveness regarding ECA evidence as mentioned within the previous theme is claimed to be caused by methodological issues [86,89]. Methodological issues make it difficult to compare study results and to draw generic conclusions. One of those issues is the variation among ECAs. To name a few:

- Different modalities used for output: (synthesized or natural) speech or text
- Different levels of responsive emotional behavior: from textual responses projected alongside a static ECA to fine-grained ECA facial expressions intended to mirror the user’s facial expressions
- Different roles: tutor, peer, interviewer, coach
- Different implementations/different computer code applied as artificial intelligence to steer the ECA

Many of these issues can be resolved by using a common, open research platform for ECAs, such as the Virtual Human platform (as provided by University of Southern California (USC) and the Institute for Creative Technologies (ICT), Los Angeles, USA; see also [87]). Other issues can potentially be resolved by a common design framework for ECAs as proposed by Veletsianos et al with their EnALI framework [88].

Concerning the duration of the change programs, several studies (eg, [66,67]) stress that the majority of virtual coaching studies concern short time spans of tens of minutes, which makes it difficult to study the development of the human-computer relationship and to realize effects on user behavior. Both Bickmore et al and Creed et al [66,67] conducted emotional virtual coach studies that spanned more than 28 days. They both found that deploying the emotional ECA did not result in user behavior changes but that users in general preferred to interact with the emotional virtual coaches.

Altogether Dehn and van Mulken [86] summarize the situation as follows: “the simple question as to whether an animated interface improves human-computer interaction does not appear to be the appropriate question to ask. Rather, the question to ask is: what kind of animated agent used in what kind of domain influence what aspects of the user’s attitudes or performance.”

## Discussion

### Principal Findings

#### Research Questions

Part 1 of this scoping review addressed the following research question:

Is there a set of generic user support needs that are currently not sufficiently addressed within eHealth interventions requiring repeated use that may result in a lower user experience and therefore lower user adherence?

We found various user needs and issues related to support, which we divided into the following two main categories:

- *Task-related system support*; concrete performance-related feedback
- *Emotion-related system support*; support that has an empathic nature

It appeared that both task-related support and emotion-related support are regularly expressed user needs. Both needs therefore merit further attention in terms of research that aims to improve user adherence.

Part 2 of this scoping review addressed the following research question:

What are main supportive features of ECAs that could potentially address user support needs?

Information was scarce and a direct answer to this question could not be found. However, we were able to find relevant information on the ECA features of *multimedia*, *relationship*, and *usefulness for eHealth adherence*.

Furthermore, we made two distinctions:

- *Nonresponsive (autonomous) ECAs*. These ECAs are not designed with the intention to capture and respond to emotionally expressed user needs. These kinds of ECAs have demonstrated that they can engage users. These ECAs run the risk of annoying the user and will then become counterproductive.
- *Responsive ECAs*. These ECAs are designed with the intention to respond to user needs in real time. These ECAs have the capacity to detect and process verbal and nonverbal information uttered by humans. However, realization of these ECAs is a heavy task, requiring costly computational modeling of user BDI [77] and affective loop facilities with a high chance of failure.

Table 4 associates the needs from part 1 with the themes addressed within part 2 and indicates whether responsive or nonresponsive ECAs can address the user need.

#### Nonresponsive ECAs

As described within Table 4, nonresponsive ECAs can provide task-related support such as setting and reviewing log-in goals and emotion-related support by the delivery of scheduled supportive messages (need 1). Furthermore, nonresponsive ECAs are capable of motivating users by techniques such as praising (need 5), performance feedback (need 6), and setting expectation levels toward user (need 8). Altogether, nonresponsive ECAs are likely capable of helping out users with more straightforward motivational tasks.

#### Responsive ECAs

In contrast to nonresponsive ECAs, responsive ECAs are capable of performing more complex motivational tasks as described within the needs 3, 4, and 7. First, responsive ECAs are capable of having a dialogue with the user during which concrete daily issues the user is facing can be effectively discussed (need 3). Further research should focus on effective countermeasures for users losing interest interacting with responsive ECAs during longer-term interactions (eg, 4-10 weeks with daily contact) [65]. Second, during a dialogue with the ECA, the user can share experiences as an addition to filling in a questionnaire. This provides the user with the ability to refine the communication process (need 4). Further research should focus on the accompanying technical and conversational complexities of such a refining dialogue. Third, a responsive ECA is capable of assisting users who cope with experiences of negative affect during their change process (need 7). However, current experimental setups can only artificially create moments of frustration. Further research should focus on ECAs that detect and respond to spontaneous user emotion.

#### Not Addressable by Either Responsive or Nonresponsive ECAs

Dialogues between the user and ECA on deep, personal issues (need 2) are currently technically too complex to realize. Smooth interactions are a necessary condition for ECAs to become and remain a trustworthy counterpart. None of the ECAs found are capable of truly meeting this condition of smoothness. As a result of future progress within the artificial intelligence field, this may change for the better. For the moment, these dialogues should be best carried out by a human support provider.

**Table 4:** User needs with supportive elements, associated embodied conversational agent (ECA) features, and the needed level of responsiveness of the ECA.

User need or issue	Supportive element	Associated ECA features	Needed responsiveness
1. Overcome users’ feelings of isolation	*Task-related support* can fulfill this need by setting and reviewing log-in goals *. Emotion-related support* can fulfill this need by establishing a supportive relationship,	Computers as social actors; visible conversation partner; human-computer relationship	A nonresponsive embodied conversational agent (ECA) is sufficient
2. Deeper interest in the user’s situation	*Emotion-related support* can fulfill this need by providing the user with the opportunity to talk about the impact of the disease on having become a patient.	Computers as social actors; open dialogue; visible conversation partner; human-computer relationship; responsive verbal and nonverbal communication	No ECA is currently likely to be able to address this user need
3. Interest in fundamental daily issues the user is struggling with	*Emotion-related support* can fulfill this need by asking the users about their daily experiences and issues.	Computers as social actors; open dialogue; visible conversation partner; human-computer relationship; responsive verbal and nonverbal communication	A responsive ECA is necessary; further research is advised
4. The ability for the user to refine the communication process	*Emotion-related support* can be provided alongside a more open interaction between the user and the system.	Open dialogue	A responsive ECA is necessary; further research is advised
5. The user’s need for encouragement	*Emotion-related support* could be delivered in terms of, for example, praising the user.	Motivational effects	A nonresponsive ECA is sufficient
6. Performance feedback mechanism for user responses	*Task-related support* can fulfill this need by reviewing the user’s contributions and by providing corrections in case the user made factual errors.	Computers as social actors; visible conversation partner; human-computer relationship	A nonresponsive ECA is sufficient
7. Users coping with experiences of negative affect during their change process	*Emotion-related support* can be extended in the sense of providing a dose of positive affect at the right moment.	Responsive verbal and nonverbal communication; motivational effects	A responsive ECA is necessary; further research is advised
8. Creating a setting of accountability toward the user	*Task-related support* can play a positive role by objective goal setting.	Computers as social actors; human-computer relationship	A nonresponsive ECA is sufficient

#### Design Factors for Both Responsive and Nonresponsive ECAs

The ECA literature of part 2, for example [56,74], gave indications on successful design of ECAs. Some design factors have generic relevance, irrespective of deploying either a responsive or a nonresponsive ECA within an eHealth intervention. First, it is recommended [74] that the ECA communicates its *intention*, *capabilities*, and *limitations*. That is, the ECA presents itself (eg, as a coach, tutor, or peer) before the start of the intervention and behaves according to its role consistently. This way, the user will have clear expectations of the ECA’s role. Second, users should have control over the presence of the ECA, especially during longer-term interactions. This will avoid user annoyance as reported by Bickmore et al [17]. Third, it is recommended that the ECA has short dialogues with the user. Systems that permit longer open-ended dialogues are playfully tested [52]. By limiting the scope and length of the dialogues, the ECA will more likely keep up its credibility.

### Limitations

This review has several limitations. Due to the nature of this study as a scoping review, no quantitative analyses were done, and selection of the studies was done by interpretation of the researchers. No exclusion criteria were applied with regard to the quality of the studies to ensure broad coverage of the studied topics.

As we looked for generic user support needs in part 1, we did not take the type of mental and/or physical disorder into account. In addition, we left out factors such as user personality. The rationale was to separate the subject of user experience from the user’s characteristics, but it is not certain that this separation always holds. Although we included user experience in our search string, we left out more fine-grained search terms, such as for user-centered design, to keep the search focused on the core issues. This focus on generic user needs has resulted in a broad overview of the needs and the possibilities of ECAs to address these needs, but when designing Web-based intervention for a specific target group, more research is needed to understand their specific needs for support.

Within part 2, we were aiming for on-screen solutions that could be added to the eHealth environments in *practice.* As a result, we left out studies on human-robot interaction (requiring an off-screen robot), studies with a focus on Wizard-of-Oz solutions (during which scholars steer the ECAs), and studies on virtual reality (requiring special glasses). We do not mean to imply that these technologies are not potentially interesting but only that they are less practical in the context of Web-based interventions.

### Conclusions

We conclude that users of self-guided eHealth interventions can likely profit from the support of *nonresponsive* ECAs for small motivational issues. Nonresponsive ECAs that explicitly express their supportive intention and act accordingly make self-guided eHealth environments a more user-friendly experience. This is likely to pay out in terms of adherence.

*Responsive ECAs* are expected to be capable of dealing with more profound motivational issues. This will require a sophisticated technological design, with sensors to capture user emotions in real time, artificial intelligence for interpretation, and speech facilities for smooth replies. The concepts of assessing a user’s BDI and of deploying the affective loop to resolve user frustration are intriguing. They fit with the concept of counteracting ego depletion as addressed within part 1, theme 7.

Responsive ECAs are also relevant from other perspectives. Psychological experiments extensively make use of questionnaires to gather user data. As touched upon in part 1, need 4, questionnaires are structured yet limited communication tools by design. The ECA’s sensors that deliver real-time signals on the user’s BDI during experiments can provide an additional source of user information for analysis. As an alternative to the sensor and artificial intelligence technology working in real time, logs on intervention usage (eg, number of log-ins, time in between log-ins) could predict lower user motivation.

To successfully *motivate* the user, the ECA should make use of relationship theories. The social exchange theory suggested by Krämer et al (see Table 3) provides a promising example. Application of this theory to eHealth suggests that humans prefer *equitable* human-computer relationships in which the contribution of rewards and costs are roughly equal. For an eHealth intervention, a dose of positive encouragement may serve as an effective counterbalance to the user’s effort invested. Put differently, where the eHealth intervention not only *demands* but also *provides* support, the human-computer relationship may be more equitable. Such an equipollent relationship will hypothetically last longer, as is exemplified among humans. Reversely, research on human-to-human relationship theories can profit from research on responsive ECAs, that is, an ECA that bases its acts on a human-to-human relationship theory makes this theory potentially verifiable. Testing the effects of the ECA on a user can contribute to a deeper understanding of the relationship theory.

**Figure 3:**
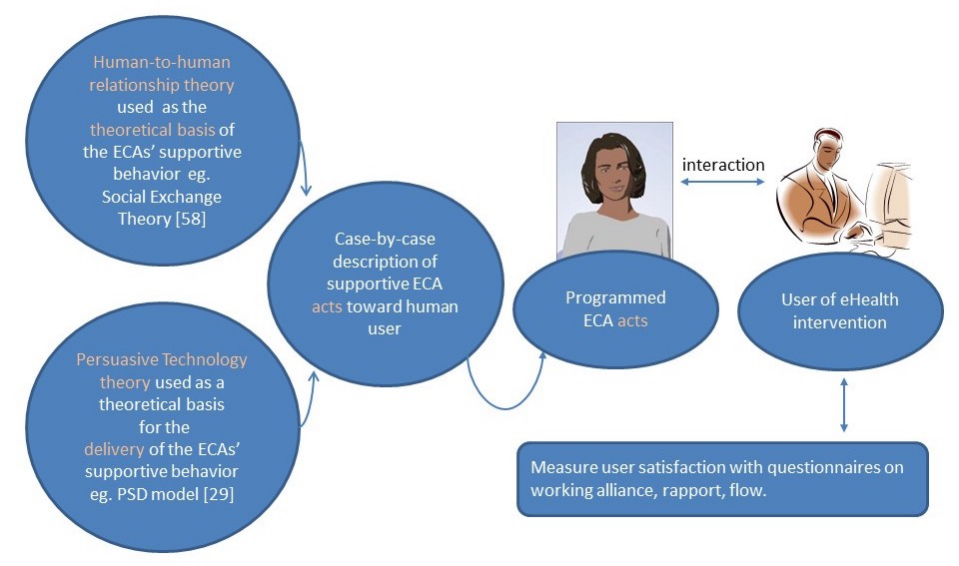
Proposal for a theory-based framework for supportive electronic health (eHealth) embodied conversational agents (ECAs).

Finally, we would like to propose a research framework. Following the advice of Dehn and van Mulken [86] to be specific about ECAs, our framework describes a *supportive ECA as an adjunct to an eHealth or ITS solution*. As Figure 3 depicts, the ECA is theory based, both from a *relationship* perspective and from a *persuasive technology* perspective. These theories lead to *supportive* ECA *acts*, as realized during the *programming phase*. When running an experiment on ECA-user interaction, the user’s level of satisfaction is measured. This is done *postexperimentally* by means of questionnaires. Additionally, *intermediary* user signals (not depicted) can be captured by sensors and analyzed. This information can be fed into the responsive ECA to make its behavior adaptive. It can also be used to cross-check the questionnaires. As expressed by Michie et al [90], eHealth interventions should be theory based. We would like to add, so should ECAs that support their users.

## Appendix

Multimedia Appendix 1 Keywords search part 1 and 2.

## Abbreviations

BDI: belief, desire, and interest
ECA: embodied conversational agent
eHealth: electronic health
ITS: intelligent tutoring system
