# Supplementary material for: Self-Guided Web-Based Interventions: Scoping Review on User Needs and the Potential of Embodied Conversational Agents to Address Them
Source: J Med Internet Res. 2017 Nov 16;19(11):e383. doi: 10.2196/jmir.7351 (PMC5709656; doi:10.2196/jmir.7351)
Supplement: Multimedia Appendix 1 [file jmir_v19i11e383_app1.pdf]

Appendix 1: Keywords search part 1 & 2

1.
During **part 1** a selection of studies was made within the Scopus database, based on the following query on title, abstract and keywords:
(motivator OR support OR "user experience" OR engage)
AND ("web-based intervention" OR "online intervention" OR "computerized therapy" OR "online CBT" OR "digital therapy" OR e-health)
AND (meta OR "systematic review" OR "qualitative study")
AND NOT (medication))

2.
During **Part 2** a selection of studies was made within the Scopus and Web of Science database, based on the following query on title, abstract and keywords:
("virtual agent" OR "virtual human" OR "embodied conversational agent")
AND (web-based intervention OR e-health OR "Intelligent Tutoring System" OR "ITS")
AND (adherence or support OR empathy OR alliance OR "user experience" OR persuasive OR rapport)
